# Construction and Analysis of a Diagnostic Model Based on Differential Expression Genes in Patients With Major Depressive Disorder

**DOI:** 10.3389/fpsyt.2021.762683

**Published:** 2021-12-09

**Authors:** Qing Long, Rui Wang, Maoyang Feng, Xinling Zhao, Yilin Liu, Xiao Ma, Lei Yu, Shujun Li, Zeyi Guo, Yun Zhu, Zhaowei Teng, Yong Zeng

**Affiliations:** ^1^Sixth Affiliated Hospital of Kunming Medical University, Yuxi, China; ^2^Institute for Health Sciences, Kunming Medical University, Kunming, China; ^3^First Affiliated Hospital of Kunming Medical University, Kunming, China; ^4^First People's Hospital of Yunnan Province, Kunming, China

**Keywords:** major depressive disorder (MDD), bioinformatical analysis, differentially expressed genes (DEG), integrated analysis, diagnostic model

## Abstract

**Background:** Major depressive disorder (MDD) is a common and severe psychiatric disorder with a heavy burden on the individual and society. However, the prevalence varies significantly owing to the lack of auxiliary diagnostic biomarkers. To identify the shared differential expression genes (DEGs) with potential diagnostic value in both the hippocampus and whole blood, a systematic and integrated bioinformatics analysis was carried out.

**Methods:** Two datasets from the Gene Expression Omnibus database (GSE53987 and GSE98793) were downloaded and analyzed separately. A weighted gene co-expression network analysis was performed to construct the co-expression gene network of DEGs from GSE53987, and the most disease-related module was extracted. The shared DEGs from the module and GSE98793 were identified using a Venn diagram. Functional pathway prediction was used to identify the most disease-related DEGs. Finally, several DEGs were chosen, and their potential diagnostic value was determined by receiver operating characteristic curve analysis.

**Results:** After weighted gene co-expression network analysis, the most MDD-related module (MEgrey) was identified, and 623 DEGs were extracted from this module. The intersection between MEgrey and GSE98793 was calculated, and 163 common DEGs were identified. The co-expression network of 163 DEGs from these was then reconstructed. All hub genes were identified based on the connective degree of the reconstructed co-expression network. Based on the results of functional pathway enrichment, 17 candidate hub genes were identified. Finally, logistic regression and receiver operating characteristic curves showed that three candidate hub genes (*CEP350, SMAD5*, and *HSPG2*) had relatively high auxiliary value in the diagnosis of MDD.

**Conclusion:** Our results showed that the combination of *CEP350, SMAD5*, and *HSPG2* has a relatively high diagnostic value for MDD. Pathway enrichment analysis also showed that these genes may play an important role in the pathogenesis of MDD. These results suggest a potentially important role for this gene combination in clinical practice.

## Introduction

Major depressive disorder (MDD) is a common mental disorder characterized by the presence of a set of depressed symptoms for at least 2 weeks (1). MDD places a heavy burden on medical systems worldwide (2, 3); however, the prevalence of this disorder varies geographically. In China, 3.02% of the population suffers from MDD (4). Smith argued that this relatively low morbidity may be due to different diagnostic criteria used in China (4). The diagnosis of major depression is based on clinical symptoms. Thus, it is difficult to reach a consensus on the symptoms due to the varied recognition of symptoms among individual clinicians and in different cultures. In addition, the lack of biomarkers contributes to regional differences in MDD diagnosis. Consequently, it is critical to identify biomarkers with reference values to assist in objective clinical identification.

The mechanisms underlying MDD remain unclear, and many hypotheses have been controversial. Historically, the monoamine hypothesis has been the most acceptable (5, 6); however, Boku et al. (7) argued that neuroplasticity and neurogenesis hypotheses should also be considered. In addition, a growing body of evidence suggests that inflammatory mechanisms also play a crucial role in the pathophysiological processes of MDD (8, 9). It has also been reported that there are some differences in gene expression between patients with MDD and normal individuals (10). Indeed, the impact of genes on the development of this disease has also been emphasized (11–13). Despite these various hypotheses, these studies have failed to provide specific or stable biomarkers that could be used for the diagnosis of MDD.

According to Mahajan et al. (14), compared to psychiatrically normal control subjects, some neuro-inflammatory genes in the hippocampus of MDD patients are differentially expressed. A recent study found that the gene expression pattern of Brodmann's area 9 (424 genes), 10 (52 genes), and 33 (59 genes) is altered between MDD and non-affected controls (15). Moreover, Mamdani et al. found that interferon regulatory factor 7 (IRF7) was upregulated by citalopram treatment *via* comparing the individuals with or without good response to the treatment (16). Thus, it has been shown that the different gene expression pattern in brain areas may have potential to distinguish MDD and psychiatrically-normal subjects and even the antidepressant response. However, these results are with few values in application to clinic practice because of the difficulty and immorality in obtaining brain samples from alive MDD patients. Therefore, the differences of gene expression pattern between MDD patients and non-psychiatric persons in periphery blood seems valuable for psychiatrists and the researchers of this field. Leday et al. (9) also identified that gene expression in the whole blood of MDD patients was different from that in non-psychotic individuals. Recently, a quantitative review has revealed that the transcriptional changes between MDD and non-affected controls were significantly different, especially in innate immune-related genes (17). Besides, a growing body of studies also showed that some peripheral miRNAs may potentials act as biomarkers for major depression and antidepressant treatment response (18). Consequently, it can be proposed that the differences of peripheral transcription between MDD patients and non-psychotic individuals may be a sign of diagnostic and treatment response markers. Despite numerous studies reporting that gene expression is altered in people with MDD, few studies have attempted to identify consistent differentially expressed genes (DEGs) between different tissues, especially between the peripheral blood and the brain.

In this study, we aimed to identify the shared DEGs with potential diagnostic value in both the hippocampus and the whole blood using a systematic and integrated bioinformatics analysis.

## Materials and Methods

### Data Source

We downloaded two datasets (GSE53987 and GSE98793) from the Gene Expression Omnibus database. Both datasets were based on the Affymetrix Human Genome U133A array. There were a total of 17 samples of post-mortem hippocampal tissue from MDD patients in GSE53987. In addition, 18 post-mortem hippocampal tissue samples from healthy controls were included in this dataset. The GSE98793 dataset comprised 64 controls and 128 MDD patients. The MDD patients included those with and without generalized anxiety disorder (64 patients in each group). We analyzed data from MDD patients and those who were free of anxiety disorders.

### Identification of DEGs and Construction of Co-expression Network

The online tool, GEO2R, was used to analyze the two datasets. This tool is based on R language investigation (19). After defining the control and MDD groups, we compared the two groups. The combination of *P*-value and |logFC| are a typical method which is utilized by many studies (20, 21). Besides, because of the high heterogeneity between hippocampus and periphery blood, a relatively broaden threshold is needed to find more probable consistent DEGs. Thus, we defined significant up-regulation genes as those with LogFC > 0 and *P* < 0.05. On the contrary, significant down-regulated genes were defined as those with LogFC < 0 and *P* < 0.05. In other words, we artificially defined a value < 0.05, and |LogFC| > 0 as the cutoff criteria to judge the DEGs. The WGCNA package in R platform (22) was used to construct the co-expression network of the DEGs in GSE53987. The preservation of the constructed modules was judged by Z-summary which was shown in a previous study (23). After calculating and filtering, disease-related modules were loaded. Then, by scanning and comparing the correlation coefficients, the most relevant MDD module was extracted.

### Identification of the Shared DEGs and Reconstruction of the Co-expression Network

To find the shared DEGs between the most MDD-related modules and GSE98793, an online Venn diagram tool was used (http://www.ehbio.com/test/venn/). We then obtained the shared DEGs and extracted the co-expression network based on the selected module. Next, we used an application named Cytoscape (version 3.7.1) to reconstruct the co-expression network. A Cytoscape plugin known as cytoHubba was used to select DEGs with a relatively high degree of connectivity (defined as hub genes) from the complex network (24). CytoHubba plugin includes 12 algorisms, only degree algorism was used in this process.

### Pathway Enrichment Analysis and Screening Out the Candidate Genes Used for the Diagnosis of MDD

Kyoto Encyclopedia of Genes and Genomes pathway enrichment and Gene Ontology analysis were performed to explore the potential molecular mechanisms of the DEGs in the extracted module in the neuropsychiatric process. These two analytical tools are available on the website (https://metascape.org/gp/index.html). The Gene Ontology project contains three clusters: biological processes (such as metabolic processes and immune system processes), cellular components (for example, synapse and protein-containing complex), and molecular function (the genes biological activities, such as structural activity and transporter activity). The Kyoto Encyclopedia of Genes and Genomes project is used for enrichment of genes in diseases and organismal systems. Finally, DEGs enriched in the pathway of interest were included in further processing.

### Potential Diagnostic Value Identification of the Candidate Hub Genes

The expression submatrix was obtained from two datasets. To explore the potential diagnostic value of selected candidate hub genes, a series of statistical analysis was used. Firstly, to construct an eligible combination model, logistic regression analysis was performed based on the expression selected hub genes in both datasets. After acquiring the optimal model, receiver operating character (ROC) curve was used to evaluate the potential diagnostic efficiency of it in both datasets. Finally, nomogram analysis and calibration curve were applied for identify prediction accuracy and risk evaluation of the combination model.

### Statistical Analysis

To draw the receiver operator characteristic (ROC) curve, we downloaded the submatrices of the two datasets. The expression of DEGs was used to identify potential diagnostic values. All statistical analyses were performed using the R software (version 4.1.0; R Foundation for Statistical Computing, Vienna, Austria). The pROC package (version 1.17.0.1) was used to calculate the potential diagnostic value of the shared hub genes in both datasets. Statistical tests resulting in a *p*-value < 0.05 were considered statistically significant.

## Results

### DEGs Common to Whole Blood and Hippocampus Samples

The gene chip, GSE53987, contains the gene expression of three regions of post-mortem brain tissue samples from patients with MDD and non-psychiatric individuals. Gene expression profiles from the hippocampus were extracted. A total of 45,783 genes were tested using GEO2R. After scanning and filtering those without matched gene symbols, 1,280 genes were identified as DEGs. Another Gene Expression Omnibus dataset, GSE98793, consisted of gene expression profiles from the whole blood of 128 persons, including 64 MDD patients and 64 healthy controls. Using the same tool and criteria, 3864 DEGs were identified.

**Figure 1:**
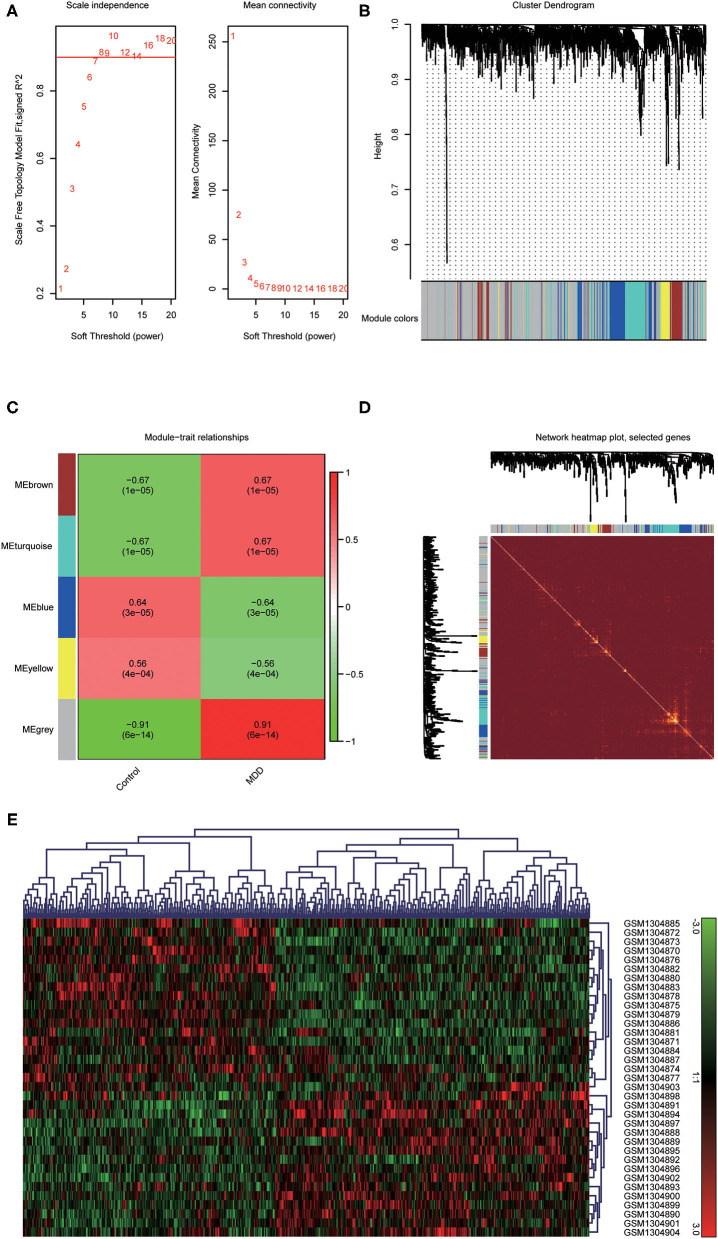
Construction of a weighted gene co-expression network analysis. **(A)** Selection of soft threshold; **(B)** Co-expression network of differential expressed genes from GSE53987; **(C)** Disease-related module: the words at the top of every module refer to the correlation index, and the words in brackets refer to the *p*-value; **(D)** The heatmap of the correlationship between modules of the selected genes; **(E)** The clustered gene expression heatmap of MEgrey module.

**Figure 2:**
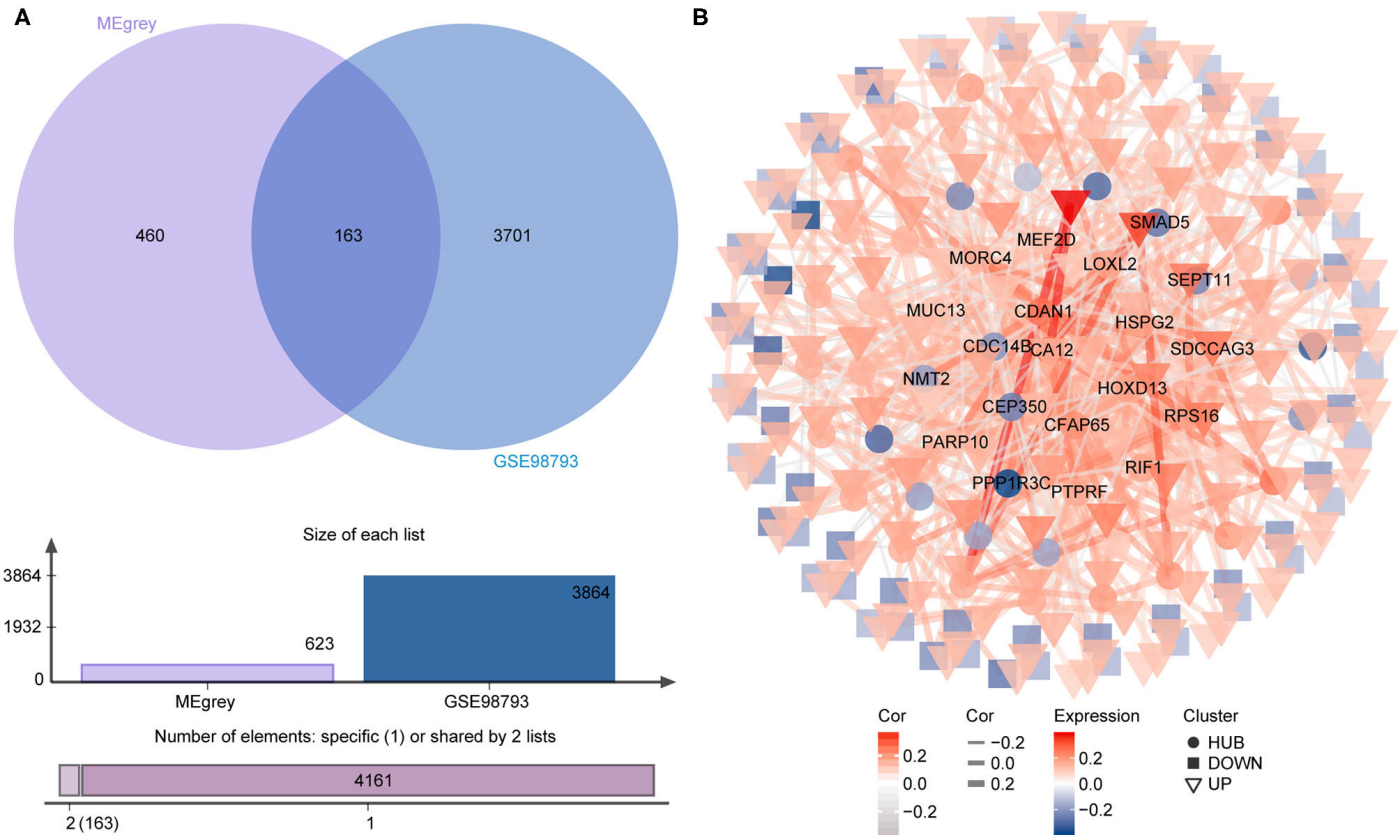
The differential expressed gene intersection between MEgrey and GSE98793. **(A)** The calculation progression of intersection between MEgrey and GSE98793; **(B)** The reconstruction of the co-expression differential expressed gene network from MEgrey module.

To identify the most disease-related DEGs from GSE53987, we performed a weighted gene co-expression network (Figure 1) analysis to construct the co-expression network. The module-trait analysis showed that the MEgrey module was most related to MDD (*r*^2^ = 0.91, *p* < 0.001). Furthermore, there was a significant negative correlation between this module and the healthy controls. Thus, this module has the greatest potential to distinguish patients with MDD from healthy controls. The preservation test of modules also showed that MEgrey was a high preserved module with a Z-summary score > 20 (data was shown in Supplementary Figure S1). After extracting the MEgrey module, 623 DEGs were studied further. To find the DEGs in common between the hippocampus and whole blood, online Venn diagrams were constructed. In addition, 163 DEGs were identified in both datasets (Figure 2A).

### Reconstruction of the Co-expression Network of the Common DEGs and Identification of Candidate Genes

**Figure 3:**
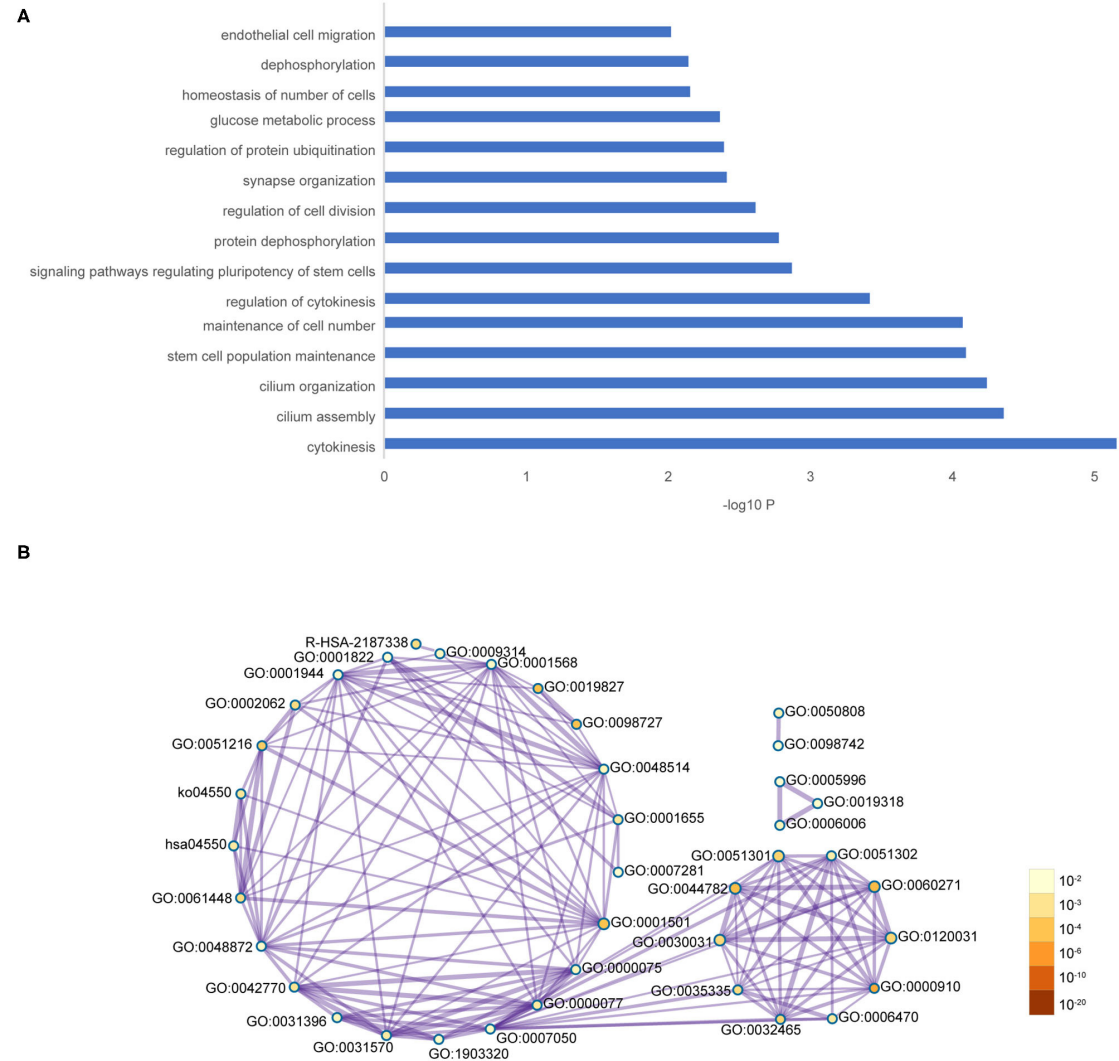
Functional pathway enrichment of hub genes. **(A)** The interested pathway. **(B)** All of the enrichment pathway terms of hub genes.

The co-expression network of the shared 163 DEGs was reconstructed using the MEgrey module. Then, the network was introduced to Cytoscape and genes with a connective degree ≥ 4 were defined as hub genes utilizing the cytoHubba plugin. In addition, igraph and ggraph mapping were performed to optimize the reconstructed co-expression network (Figure 2B). And the separated edges and nodes were deleted. Pathway enrichment analysis was used to identify candidate DEGs. The results showed that the hub genes were enriched in cytokinesis, synapse organization, skeletal system development, and so on. After comparing the literature and existing theories, the hub genes enriched in the neuropsychiatric pathway were identified (Figure 3). A total of 17 candidate genes were included in the evaluation of potential diagnostic value. The details of these genes are listed in Table 1.

**Table 1:** List of gene hubs based on pathway enrichment.

**Gene**	**Degree**	**Pathway enrichment**	**log P**
PARP10	25	Regulation of protein ubiquitination	−2.39345
		Regulation of protein modification by small protein conjugation or removal	−2.21863
CDC14B	20	Cytokinesis	−5.15621
		Cilium assembly	−4.36233
		Cilium organization	−4.24352
		Cell division	−3.49007
		Plasma membrane bounded cell projection assembly	−3.42927
		Regulation of cytokinesis	−3.42001
		Cell projection assembly	−3.37753
		Peptidyl-tyrosine dephosphorylation	−3.23627
		Protein dephosphorylation	−2.77948
		Regulation of cell division	−2.61501
		Dephosphorylation	−2.14304
LOXL2	17	Stem cell population maintenance	−4.09643
		Maintenance of cell number	−4.07354
		Skeletal system development	−3.98426
		Cartilage development	−3.70054
		Connective tissue development	−3.23858
		Chondrocyte differentiation	−3.23627
		Blood vessel morphogenesis	−2.39957
		Blood vessel development	−2.20376
		Vasculature development	−2.12934
		Endothelial cell migration	−2.02071
PTPRF	15	Peptidyl-tyrosine dephosphorylation	−3.23627
		Protein dephosphorylation	−2.77948
		Dephosphorylation	−2.14304
		Synapse organization	−2.41323
		Cell-cell adhesion *via* plasma-membrane adhesion molecules	−2.04193
SMAD5	15	Skeletal system development	−3.98426
		Cartilage development	−3.70054
		Connective tissue development	−3.23858
		Signaling pathways regulating pluripotency of stem cells	−2.87165
		Signaling pathways regulating pluripotency of stem cells	−2.75235
		Homeostasis of number of cells	−2.15487
		Urogenital system development	−2.804
		Germ cell development	−2.1037
		Kidney development	−2.01651
SDCCAG3	13	Cytokinesis	−5.15621
		Cilium assembly	−4.36233
		Cilium organization	−4.24352
		Cell division	−3.49007
		Plasma membrane bounded cell projection assembly	−3.42927
		Regulation of cytokinesis	−3.42001
		Cell projection assembly	−3.37753
		Regulation of cell division	−2.61501
HSPG2	13	Blood vessel morphogenesis	−2.39957
		Blood vessel development	−2.20376
CEP350	11	Cilium assembly	−4.36233
		Cilium organization	−4.24352
		Plasma membrane bounded cell projection assembly	−3.42927
		Cell projection assembly	−3.37753
RIF1	10	Stem cell population maintenance	−4.09643
		Maintenance of cell number	−4.07354
		Signaling pathways regulating pluripotency of stem cells	−2.87165
		Signaling pathways regulating pluripotency of stem cells	−2.75235
SEPTIN11	10	Cytokinesis	−5.15621
		Cell division	−3.49007
		Synapse organization	−2.41323
SEPTIN6	8	Cytokinesis	−5.15621
		Cilium assembly	−4.36233
		Cilium organization	−4.24352
		Cell division	−3.49007
		Plasma membrane bounded cell projection assembly	−3.42927
		Regulation of cytokinesis	−3.42001
		Cell projection assembly	−3.37753
SOX4	7	Stem cell population maintenance	−4.09643
		Maintenance of cell number	−4.07354
		Skeletal system development	−3.98426
		Blood vessel morphogenesis	−2.39957
		Blood vessel development	−2.20376
		Homeostasis of number of cells	−2.15487
		Vasculature development	−2.12934
		Signal transduction in response to DNA damage	−2.94568
		DNA damage checkpoint	−2.79357
		DNA integrity checkpoint	−2.7283
		Regulation of protein ubiquitination	−2.39345
		Cell cycle checkpoint	−2.37003
		Cell cycle arrest	−2.22369
		Regulation of protein modification by small protein conjugation or removal	−2.21863
		Urogenital system development	−2.804
		Kidney development	−2.01651
ANGPT2	6	Blood vessel morphogenesis	−2.39957
		Blood vessel development	−2.20376
		Vasculature development	−2.12934
		Endothelial cell migration	−2.02071
		Response to radiation	−2.28481
		Urogenital system development	−2.804
		Germ cell development	−2.1037
		Kidney development	−2.01651
MAPK14	6	Skeletal system development	−3.98426
		Cartilage development	−3.70054
		Connective tissue development	−3.23858
		Chondrocyte differentiation	−3.23627
		Signaling pathways regulating pluripotency of stem cells	−2.87165
		Signaling pathways regulating pluripotency of stem cells	−2.75235
		Blood vessel morphogenesis	−2.39957
		Blood vessel development	−2.20376
		Homeostasis of number of cells	−2.15487
		Vasculature development	−2.12934
		Endothelial cell migration	−2.02071
		Response to radiation	−2.28481
		Signal transduction in response to DNA damage	−2.94568
		DNA damage checkpoint	−2.79357
		DNA integrity checkpoint	−2.7283
		Cell cycle checkpoint	−2.37003
		Synapse organization	−2.41323
		Cell-cell adhesion *via* plasma-membrane adhesion molecules	−2.04193
		Glucose metabolic process	−2.36425
		Hexose metabolic process	−2.15487
		Monosaccharide metabolic process	−2.05487
DIS3L2	5	Cell division	−3.49007
		Stem cell population maintenance	−4.09643
		Maintenance of cell number	−4.07354
CDC14A	4	Cytokinesis	−5.15621
		Cilium assembly	−4.36233
		Cilium organization	−4.24352
		Cell division	−3.49007
		Plasma membrane bounded cell projection assembly	−3.42927
		Regulation of cytokinesis	−3.42001
		Cell projection assembly	−3.37753
		Peptidyl-tyrosine dephosphorylation	−3.23627
		Protein dephosphorylation	−2.77948
		Regulation of cell division	−2.61501
		Dephosphorylation	−2.14304
		Cell cycle arrest	−2.22369
SDK2	4	Synapse organization	−2.41323
		Cell-cell adhesion *via* plasma-membrane adhesion molecules	−2.04193

### Establishment of the Signature of the DEGs for MDD Diagnosis

**Figure 4:**
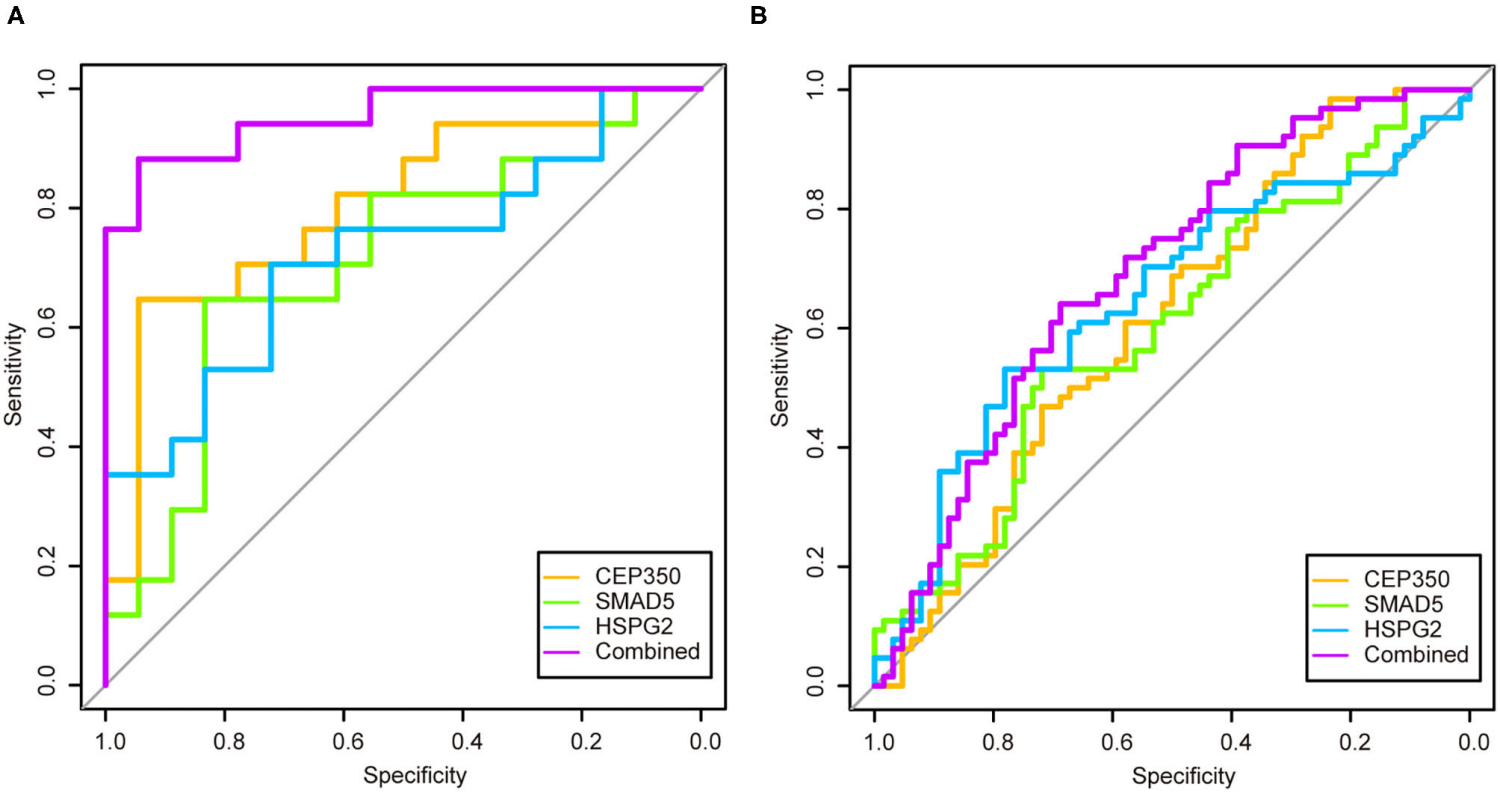
The receiver operator characteristic curves and area under the curve of the candidate genes in two datasets. **(A)** Receiver operator characteristic curves and area under the curve of the candidate genes of GSE53987; **(B)** Receiver operator characteristic curves and area under the curve of the candidate genes of GSE98793.

We used logistic regression to screen 17 candidate genes from the GSE53987. After that, three significant genes were screened out (*p* > 0.05). To validate the diagnostic role of these three gene signatures, the combination gene risk score was calculated as follows: the combination gene panel = (−0.010 × expression value of *CEP350*) + (−0.007 × expression value of *SMAD5*) + (0.022 × expression value of *HSPG2*) + 0.571. The ROC curve was used to evaluate the diagnostic value of single genes and combined genes in MDD (Figure 4A). The results showed that all three single genes had diagnostic value, and the combination gene significantly improved the diagnostic value of MDD (Figure 4B). The AUC value of the combined gene was the highest at 0.9542.

In order to verify the diagnostic value of the screened genes in MDD, we used the same method in the GSE98793 dataset. The combination gene risk score was calculated as follows: the combination gene panel = (−0.446 × expression value of *CEP350*) + (−0.139 × expression value of *SMAD5*) + (0.248 × expression value of *HSPG2*) + 3.999. The results also showed that the combined gene had a relatively high diagnostic value for MDD (Figure 4B). The AUC value of the combined gene was the largest at 0.6919.

**Figure 5:**
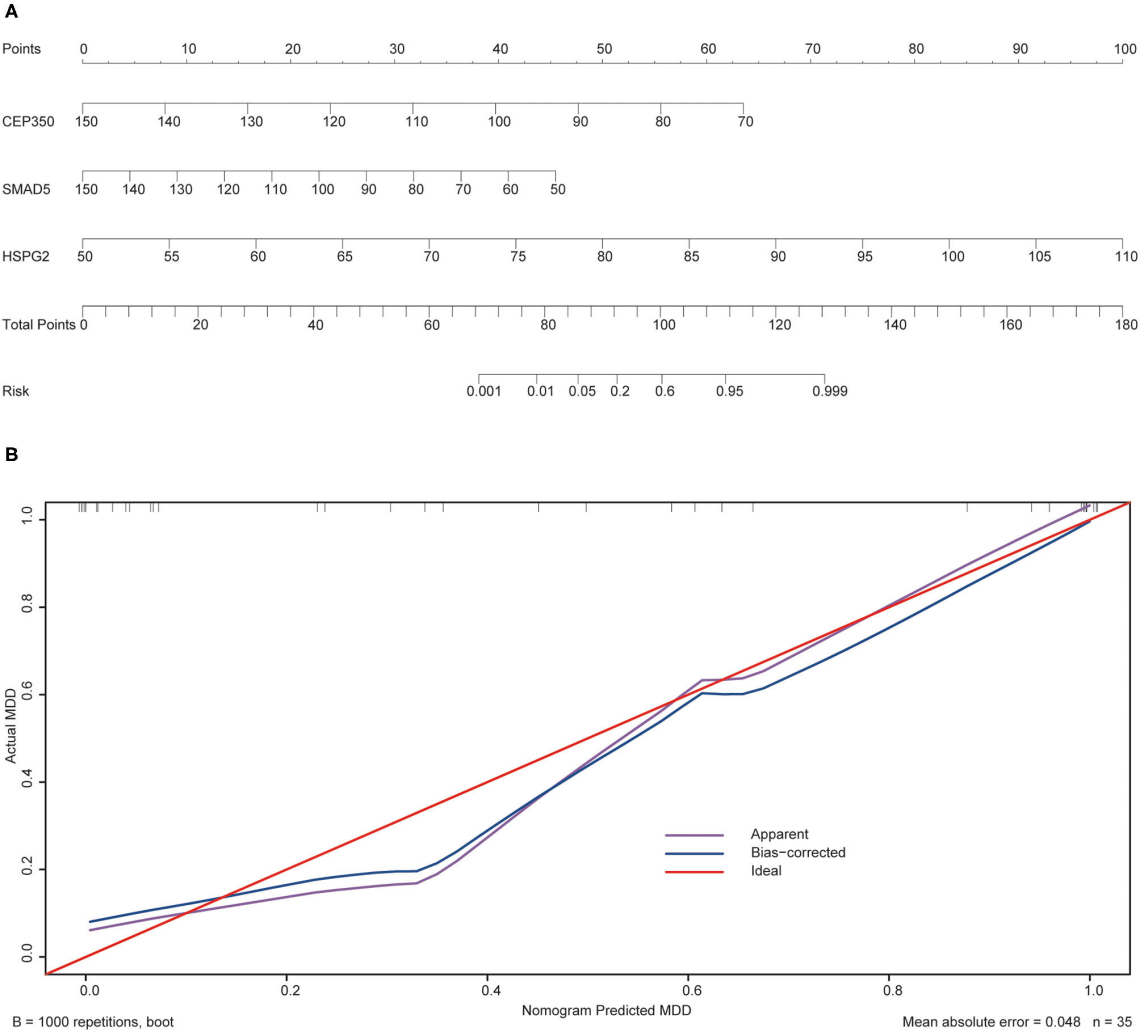
The nomogram and calibration of combination of three hub genes (CEP350, SMAD5, HSPG2) in GSE53987. **(A)** Nomogram of three hub genes: the value in points line is matched with the value in lines of the genes based on their expression. Total points line refers to the sum of the matched points of these genes. And the risk range line is matched with the corresponding range in total points line; **(B)** Calibration of combination of the three genes: the apparent line is along with ideal line, and Hosmer-Lemeshow Test showed that there is no significant difference between ideal line and apparent line.

**Figure 6:**
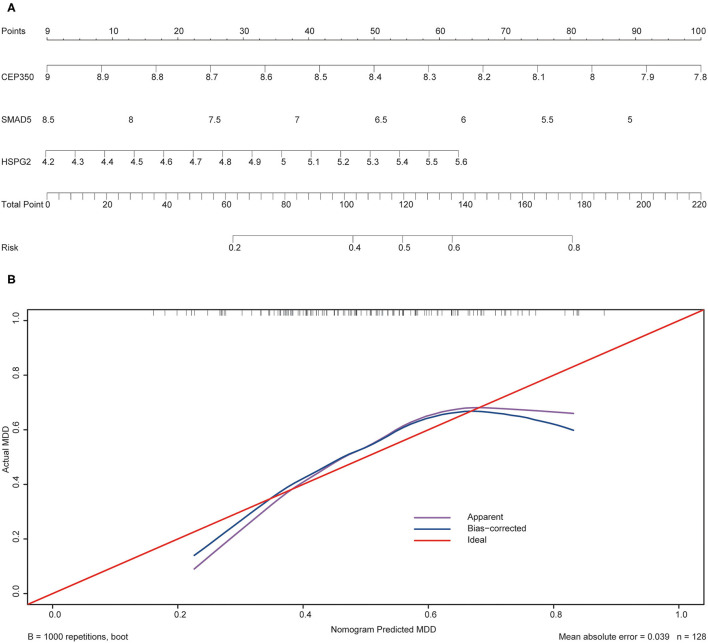
The nomogram and calibration of combination of three hub genes (CEP350, SMAD5, HSPG2) in GSE98793. **(A)** Nomogram of three hub genes: the value in points line is matched with the value in lines of the genes based on their expression. Total points line refers to the sum of the matched points of these genes. And the risk range line is matched with the corresponding range in total points line; **(B)** Calibration of combination of the three genes: the apparent line is along with ideal line, and Hosmer-Lemeshow Test showed that there is no significant difference between ideal line and apparent line.

To validate the prediction accuracy and risk evaluation of the combination genes, we draw the nomogram and calibration curve based on the expression of them. In the nomograms, each variable axis presented the value of a patient. The number of points received for the respective variable values was calculated based on an upward line. The total points axis represented the sum of the relevant numbers. And the consistency between the nomogram and the observed value was confirmed by calibration curve and Hosmer-Lemeshow test. Our calibration curves of the nomogram for risk of MDD demonstrated the prediction value is in accord with observation value in GSE53987 and GSE98793 dataset (Figures 5, 6). Hosmer-Lemeshow Test shown that *p* = 0.596 for the GSE 53987, and *p* = 0.134 for the GSE 98793, which suggested that there was no departure from perfect fit. Finally, the C-index was derived on the basis of the analysis. The C-index for the prediction nomogram was 0.961 (95% CI, 0.902–1.019) for the GSE 53987, and 0.691 (95% CI, 0.600–0.782) for the GSE 98793. These results showed a consensus with the ROC test.

## Discussion

MDD is a severe mental disorder, with a high recurrence rate (~80% of patients will have at least one recurrence in their lifetime), which places a heavy burden on individuals and on society (25). According to a global burden survey of diseases, depressive disorder ranks as the third leading cause of years lived with disability for both sexes (26). Moreover, the resistance to antidepressants is common among 30–50% of patients (18, 27, 28) with MDD (18, 27, 28). It has been found that the cure rate and prognosis of MDD partly depend on the stage. Early detection and treatment are often associated with a better response rate and prognosis (29). Moreover, a high misdiagnosis rate due to the limited knowledge of etiology and auxiliary diagnostic markers also contributes to poor recovery of patients with MDD (30, 31). Fortunately, a growing number of studies have reported that there are many alterations in gene expression between MDD patients and healthy controls in both brain and peripheral tissues (32). However, few studies have focused on the commonality of DEGs in the brain and peripheral tissues. Thus, the present study was conducted to identify the shared DEGs between the brain and peripheral blood of patients with MDD and explore their potential diagnostic value.

In the current study, DEGs from the hippocampus and whole blood of patients with MDD were analyzed. We performed a weighted gene co-expression network analysis to construct the correlation networks. The most disease-related modules were extracted. The common DEGs in both the hippocampus and peripheral blood were included after the comparison between the extracted module and the other dataset. A total of 163 common DEGs were reanalyzed from the co-expression network to identify hub genes for further study. Then, 66 hub genes based on the degree of connectivity were selected for pathway enrichment analysis. Our data showed that these hub genes were enriched in the following functional pathways: cytokinesis, cilium assembly, and cell division. These pathways have been reported to be closely related to neuropsychiatric disorders, such as dephosphorylation (33), regulation of protein ubiquitination (34), and synapse organization (35). Based on previous studies, the pathways of interest were selected and the enriched DEGs included in them were collected for the next step. After logistic regression was performed and ROC curve was calculated, three DEGs (*CEP350, SMAD5*, and *HSPG2*) with potential diagnostic value were identified as auxiliary diagnostic markers. Furthermore, the calibration curves and nomogram analysis also displayed an applicable possibility of the combination of these DEGs.

*CEP350* (centrosomal protein 350) is a key regulator of cell polarity (36) and is involved in many biological processes. It has been reported that defective ciliogenesis may result in malformations of cortical development (37). Intriguingly, a previous study showed that *CEP350* plays a role in ciliogenesis (38). Our analysis also found that *CEP350* was enriched in the cilium assembly and organization pathways. In addition, a previous study has shown that 15% of brain-expressed cilia genes were significantly different between patients with MDD and controls (39). Indeed, a genome-wide by environment interaction study with stressful life events revealed that a single nucleotide polymorphism near *CEP350* was associated with depressive symptoms in African Americans (40). Based on these findings, *CEP350* may be involved in the development of the brain and the pathogenesis of MDD.

*SMAD5* (SMAD family member 5) is well-known for its regulatory function in osteogenesis (41). The role of *SMAD5* in developmental disorders has also been revealed in recent decades. It is located at chromosome 5q31, and has been regarded as a key region associated with development. Deletion or duplication of this region results in many developmental disorders, such as developmental delay, intellectual disability, and dysmorphic features (42). Moreover, *SMAD5* has been identified as a transcription factor that participates in brain development. For instance, it plays an essential role in neuronal and glial development, and its knockdown may lead to exencephaly (43). Interestingly, it has been demonstrated that the dysregulation of brain development is strongly related to multiple psychiatric disorders, like schizophrenia (SCZ), bipolar disorder, and MDD. This suggests that *SMAD5* is potentially involved in the pathogenesis of mental diseases. Indeed, in recent studies, whole-exome sequencing has revealed that *SMAD5* is one of the candidate genes of SCZ (44) and another study showed that *SMAD5* was associated with cognitive deficits in SCZ patients (45). Thus, *SMAD5* plays an important role in neuropsychiatric disorders.

*HSPG2* (heparan sulfate proteoglycan 2) is located at chromosome 1p36, which has been identified as an essential chromosome for brain development. Multiple developmental disorders of the brain emerge with the deletion of this region, such as seizures, vision problems, hearing loss, and brain anomalies (46). Moreover, a growing number of studies have found that *HSPG2* is closely associated with tardive dyskinesia (47, 48), which is a common side effect of antipsychotics. Recent studies have also shown that *HSPG2* is closely related to neuropsychiatric disorders, and Clement et al. found that a single nucleotide polymorphism of *HSPG2* was positively associated with tardive dyskinesia occurrence in SCZ patients (49) and another study showed that a decreasing expression of *HSPG2* was observed in both frontotemporal dementia and amyotrophic lateral sclerosis (50). Moreover, *HSPG2* has been reported as a critical regulator in the maintenance and repair of the blood-brain barrier (BBB) (51). An increasing body of research has reported that BBB injuries are associated with many mental disorders, such as SCZ (52), bipolar disorder (53), and depressive disorders (54). Furthermore, it has been revealed that both chronic stress and impaired glutamate function in mouse models showed a depressive-like phenotype with downregulation of HSPG2 (55). Therefore, *HSPG2* plays an important role in the pathogenesis of MDD.

Based on the above studies and our results, these three DEGs may have important functions in the mechanism of MDD. As mentioned in the previous section, we explored the potential diagnostic value of the three DEGs. It was shown that the combination of these three DEGs has a relatively high reference value in the diagnosis of MDD in both the hippocampus and peripheral blood.

There were some limitations to the present study. First, the filtration and calculation of the genes was based on bioinformatics analysis. Although these systematic methods avoid the bias of artificial selection to a certain extent, the deficiency of clinical samples and a series of experimental validations have limited their application in MDD patients. Besides, there is limited samples and insufficient datasets to find any other suitable independent cohort that can be utilized to validate our results. The specific functions of these DEGs that underpin the pathophysiological process of MDD need to be further studied in *in vitro* and *in vivo* models.

In conclusion, our results showed that the combination of three DEGs (*CEP350, SMAD5*, and *HSPG2*) has a relatively high reference auxiliary diagnostic value for MDD. Pathway enrichment analysis also revealed that these three DEGs may play a role in the pathogenesis of MDD. Thus, this combination diagnostic model of the three genes may have potential applications in the clinical practice concerning patients with MDD.

## Data Availability Statement

Publicly available datasets were analyzed in this study. This data can be found here: https://www.ncbi.nlm.nih.gov/gds/ (GSE53987 and GSE98793).

## Author Contributions

ZT and YZe have designed this study. QL has written the manuscript. RW and MF performed the statistical analysis and visualized the results. XZ and ZG formatted the reference lists. YL, XM, and LY helped to organize the visual data. YZh and SL collected and formatted the gene list. All authors contributed to the article and approved the submitted version.

## Funding

This study was supported by the National Natural Science Foundation of China (81760253 and 81960254) and the Yunnan Health Training Project of High-level Talents (L-2017021) from YZe.

## Conflict of Interest

The authors declare that the research was conducted in the absence of any commercial or financial relationships that could be construed as a potential conflict of interest.

## Publisher's Note

All claims expressed in this article are solely those of the authors and do not necessarily represent those of their affiliated organizations, or those of the publisher, the editors and the reviewers. Any product that may be evaluated in this article, or claim that may be made by its manufacturer, is not guaranteed or endorsed by the publisher.

## Supplementary Material

Supplementary Figure S1 Preservation test of all modules with significant association of MDD. **(A)** The preservation median rank, vertical axis refers to the preservation rank value; **(B)** Z-summary test for all modules, vertical axis refers to Z-summary score. Horizontal axis refers to the number of genes for both diagrams.
