# Supplementary figures and images for: Construction and Analysis of a Diagnostic Model Based on Differential Expression Genes in Patients With Major Depressive Disorder
Source: Front Psychiatry. 2021 Dec 9;12:762683. doi: 10.3389/fpsyt.2021.762683 (PMC8695921; doi:10.3389/fpsyt.2021.762683)

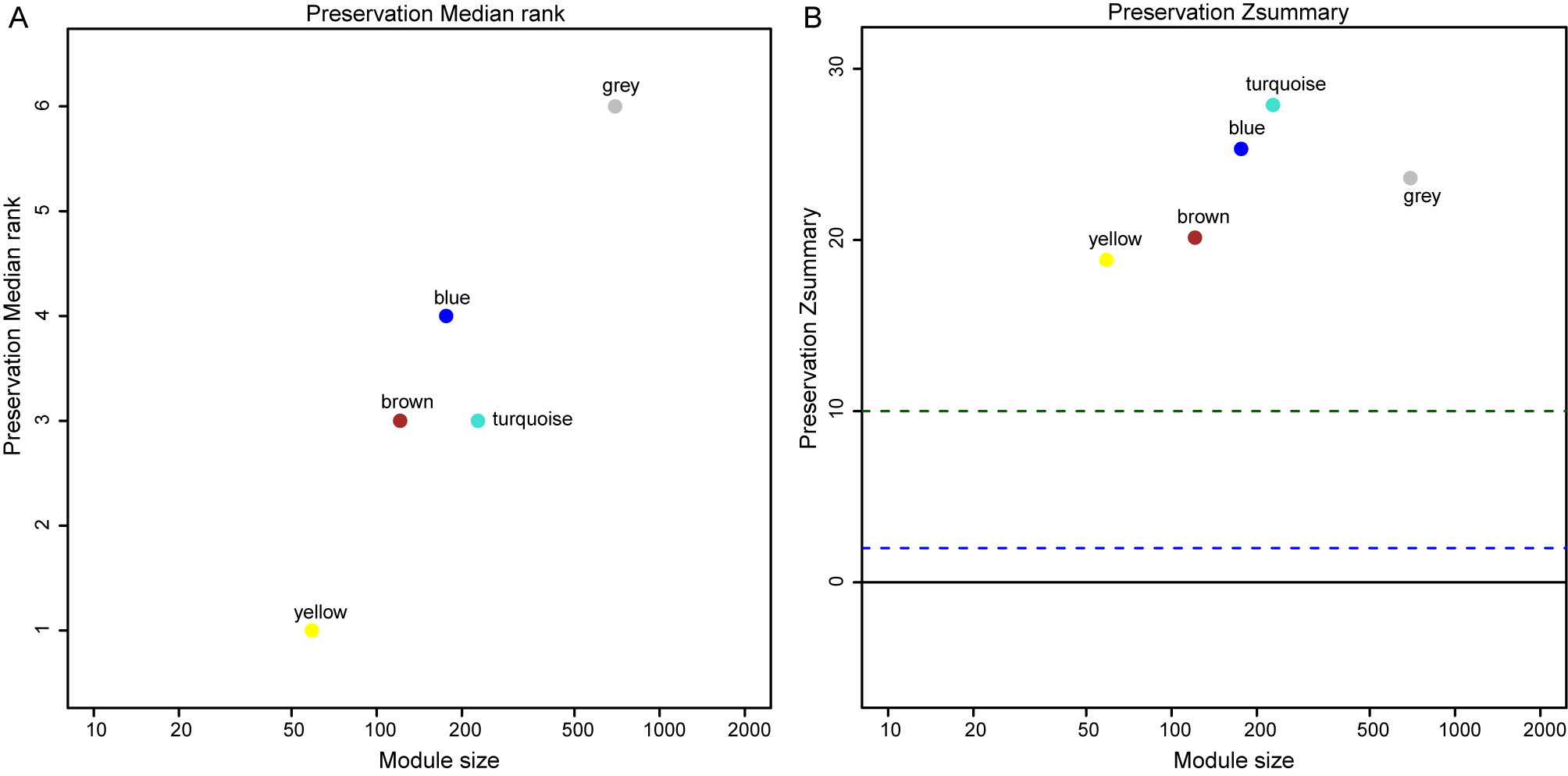

Supplement: Supplementary Figure S1 — Preservation test of all modules with significant association of MDD. (A) The preservation median rank, vertical axis refers to the preservation rank value; (B) Z-summary test for all modules, vertical axis refers to Z-summary score. Horizontal axis refers to the number of genes for both diagrams. [file Image_1.TIF]
